# A stable physisorbed layer of packed capture antibodies for high-performance sensing applications

**DOI:** 10.1039/d3tc01123b

**Published:** 2023-06-22

**Authors:** Lucia Sarcina, Cecilia Scandurra, Cinzia Di Franco, Mariapia Caputo, Michele Catacchio, Paolo Bollella, Gaetano Scamarcio, Eleonora Macchia, Luisa Torsi

**Affiliations:** a Dipartimento di Chimica, Università degli Studi di Bari Aldo Moro, Via E. Orabona 4 70125 Bari Italy luisa.torsi@uniba.it; b CNR – Institute of Photonics and Nanotechnologies 70126 Bari Italy; c Dipartimento di Farmacia-Scienze del Farmaco, Università degli Studi di Bari Aldo Moro 70126 Bari Italy eleonora.macchia@uniba.it; d CSGI (Centre for Colloid and Surface Science), Via E. Orabona 4 70125 Bari Italy; e Dipartimento Interateneo di Fisica “M. Merlin”, Università degli Studi di Bari Aldo Moro 70126 Bari Italy; f The Faculty of Science and Engineering, Åbo Akademi University 20500 Turku Finland

## Abstract

Antibody physisorption at a solid interface is a very interesting phenomenon that has important effects on applications such as the development of novel biomaterials and the rational design and fabrication of high-performance biosensors. The strategy selected to immobilize biorecognition elements can determine the performance level of a device and one of the simplest approaches is physical adsorption, which is cost-effective, fast, and compatible with printing techniques as well as with green-chemistry processes. Despite its huge advantages, physisorption is very seldom adopted, as there is an ingrained belief that it does not lead to high performance because of its lack of uniformity and long-term stability, which, however, have never been systematically investigated, particularly for bilayers of capture antibodies. Herein, the homogeneity and stability of an antibody layer against SARS-CoV-2-Spike1 (S1) protein physisorbed onto a gold surface have been investigated by means of multi-parametric surface plasmon resonance (MP-SPR). A surface coverage density of capture antibodies as high as (1.50 ± 0.06) × 10^12^ molecules per cm^−2^ is measured, corresponding to a thickness of 12 ± 1 nm. This value is compatible with a single monolayer of homogeneously deposited antibodies. The effect of the ionic strength (*i*_s_) of the antibody solution in controlling physisorption of the protein was thoroughly investigated, demonstrating an enhancement in surface coverage at lower ionic strength. An atomic force microscopy (AFM) investigation shows a globular structure attributed to *i*_s_-related aggregations of antibodies. The long-term stability over two weeks of the physisorbed proteins was also assessed. High-performance sensing was proven by evaluating figures of merit, such as the limit of detection (2 nM) and the selectivity ratio between a negative control and the sensing experiment (0.04), which is the best reported performance for an SPR S1 protein assay. These figures of merit outmatch those measured with more sophisticated biofunctionalization procedures involving chemical bonding of the capture antibodies to the gold surface. The present study opens up interesting new pathways toward the achievement of a cost-effective and scalable biofunctionalization protocol, which could guarantee the prolonged stability of the biolayer and easy handling of the biosensing system.

## Abstract 2

The uniformity and long-term stability of physisorbed antibodies against SARS-CoV-2-Spike1 at a solid interface are addressed. High-performance sensing is accomplished, outmatching the analytical performance achieved with the chemical bonding of capture antibodies.

## Graphical abstract

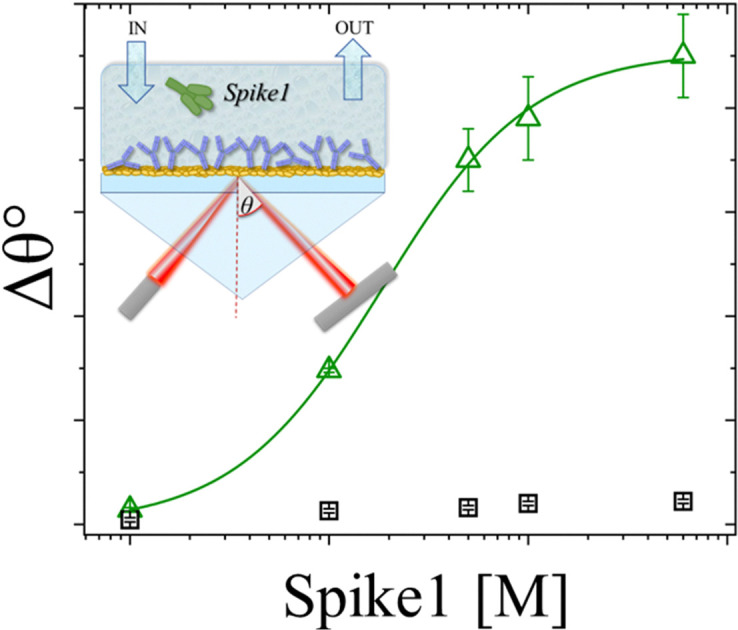

## 1. Introduction

Physisorption of antibodies or more generally of proteins onto a solid surface is a fascinating phenomenon that plays a key role in many areas of material science related to biomaterials or biosensors, but can also shine a light on fundamental protein–protein interaction phenomena regulating protein adhesion and aggregation. Despite the many different approaches that have been used to study protein physisorption, full comprehension has not yet been achieved. Some studies provide evidence that protein adsorption is an irreversible process that leads to monolayer coverage due to repulsive protein–protein interactions.^1,2^ Under physiological conditions in blood (pH = 7.4), BSA antibodies have a basic isoelectric point and are negatively charged, so that they attract serum proteins (antigens) that have an acidic isoelectric point.^3^ At high ionic strength (*i*_s_) the interaction potential is governed by an attractive potential that is weak and long-range, proving that the interactions are not screened beyond the Debye length. On the other hand, no gelation or precipitation was observed because of the concomitant increase in short-range, repulsive interaction associated with the creation of a hydration layer at higher salt concentration.^3^ The competition between long-range attractive and short-range repulsive interactions can explain the stability of BSA solution at high ionic strength.

The physical adsorption of capture antibodies, relying on intermolecular forces, mainly hydrophobic, ionic and/or van der Waals interactions,^4^ is one of the simplest and most sustainable biofunctionalization approaches, being also cost-effective, rapid,^5^ and compatible with printing techniques.^6^ Since it does not require coupling reactions along with the associated chemical modifications, it is also *green*. Despite all these advantages, physisorption has rarely been used in biosensing platforms as there is an engrained belief that a stable and high-performance biosensor should involve a conjugated layer of biological recognition elements. They are also deemed to be poorly homogeneous^7,8^ and their long-term stability is still controversial.^9^ On the other hand, bioelectronic sensors comprising a physisorbed capture layer capable of reliably and stably assaying clinically relevant biomarkers (proteins, peptides, DNA/RNA, pathogens) directly in a biofluid at the single-molecule limit have been demonstrated.^10–12^ Physisorption is also largely used for DNA and protein grafting onto organic semiconductor layers, such as pentacene, P3HT, and PEDOT in FET devices.^13^

Here, surface plasmon resonance (SPR)^14–16^ is proposed to assess the long-term stability of physisorbed antibodies on a gold detection interface for high-performance sensing applications. To this end, the capture layer is physisorbed onto the gold coating of the SPR optical element.^17,18^ The probe is the SPR optical field localized at the interface between the physiosorbed capture layer and the gold surface. The dielectric function modifications are measured as changes in the local refractive index, induced by the deposition of the physisorbed capture antibody layer or by the capture of the antigens. Both the uniformity of surface coverage and the long-term stability of physisorbed anti-SARS-CoV-2-Spike1 (anti-S1) antibodies against S1 are investigated here. The effect of the *i*_*s*_ of the antibody solution in controlling the physisorption of the protein is also addressed. Specifically, anti-S1 capture antibodies were deposited from phosphate buffer saline (PBS) solutions (pH = 7.4) at *i*_s_ of 15 mM, 163 mM and 300 mM. Interestingly, an enhancement in the anti-S1 surface coverage at lower *i*_s_ is observed, as also confirmed by atomic force microscopy (AFM) images. Moreover, the SPR assay encompassing physisorbed capture antibodies allowed us to accomplish high-performance S1 sensing, as demonstrated by a limit-of-detection (LOD) as low as 2 nM and a selectivity ratio of 0.04, competitive with other recently developed optical sensing platforms which rely on chemical grafting of the capture antibodies.^19,20^ Prolonged storage in PBS as well as long-term stability after storage in HPLC water (shelf-life) prove there is no loss of antigen binding capacity. The successful capture of S1 protein was demonstrated even after two weeks of aging, whose performance was comparable with that measured immediately after physisorption. Remarkably, the study herein proposed is extremely general, as it is applicable to a wide spectrum of human antibodies.^21^ This study opens up interesting pathways toward the achievement of a cost-effective and scalable biofunctionalization protocol, which could guarantee prolonged stability of the biolayer and easy handling of the whole biosensing system.

## 2. Materials and methods

SARS-Cov2 (2019-nCov) Spike S1 recombinant monoclonal antibody (anti-S1) expressed from HEK293 cells and SARS-CoV2 (2019-nCov) Spike S1 His Recombinant Protein (*M*_W_ 76.5 kDa) were purchased from Sino Biological and used with no further purification. Bovine serum albumin (BSA, molecular weight 66 kDa) was purchased from Sigma-Aldrich and used without further purification. Phosphate buffered saline (PBS) tablets were purchased from Merck Millipore (formerly Sigma-Aldrich). HPLC-grade water and ammonium hydroxide were obtained from Carlo Erba Reagents S.A.S. Sulfuric acid (ACS reagent, 95.0–98.0%) and hydrogen peroxide solution (H_2_O_2_ 30 wt% in H_2_O) were purchased from Sigma-Aldrich. Gold slides were cleaned in an NH_4_OH/H_2_O_2_ aqueous solution (1 : 1 : 5 v/v) at 80–90 °C for 10 min; then rinsed with water, dried with nitrogen, and treated for 10 min in a UV-ozone cleaner.

**Fig. 1:**
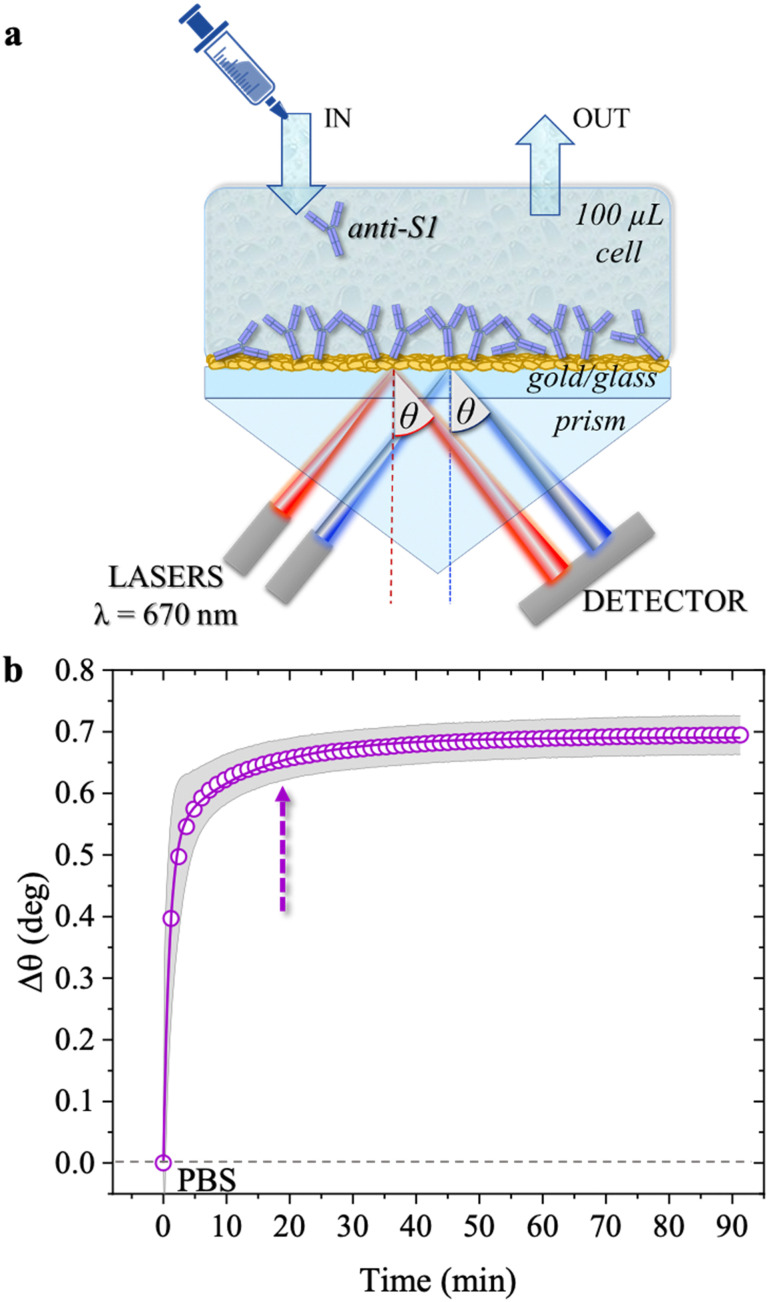
(a) Schematic representation of the SPR Navi 200-L apparatus in the Kretschmann configuration. The manual injection of a sample volume of 100 μL is performed with a sterile syringe. (b) The open symbols refer to the sensogram (Δ*θ vs*. time) registered during the physisorption of anti-S1 on the SPR gold slide. The solid line is fitted with eqn (1). The purple dotted arrow indicates the minimum time required for the biolayer to reach 95% surface coverage, namely 19 minutes.

SPR measurements were performed with a BioNavis-200 Multi-Parametric Surface Plasmon Resonance (MP-SPR) Navi^TM^ instrument, in the Kretschmann configuration where the optical signal is the SPR peak intensity/position as a function of a wide range of inspected laser incident angles, *θ*. SPR relies on the evanescent wave associated with the total internal reflection of a laser at the boundary between a higher refractive index prism and a dielectric medium (Fig. 1a).^22^ The impinging light is totally (internally) reflected at an incidence angle *θ* larger than the Brewster angle. A surface plasmon arises at the interface between the metal and the dielectric medium, aqueous solutions in this study.^23^ The optical field associated with the collective oscillation propagates along the metal–dielectric interface and exponentially decays perpendicular to the surface. Such an evanescent character gives surface specificity to the technique and allows monitoring of the 30–300 nm layer deposited at the metal/liquid interface. The multi-parametric nature of the equipment allowed for contemporaneous measurement of: (i) the transient angular shift of the plasmon peak minimum *versus* time (Δ*θ vs. t*), namely the sensogram; (ii) the SPR angular reflectivity curves as a function of *θ* (reflected intensity *vs. θ*) in the angular region range (60.00°–76.00°) where the plasmonic peak falls, namely angular scan measurement. The selected angular resolution was 0.01°, that is, 88 data points per degree. The SPR modulus was equipped with two laser sources (both set at the same wavelength of 670 nm) impinging onto areas of the specimens separated by 3 mm, to assess uniformity. The two sampled areas encompassed a surface of about 9 mm^2^. Therefore, the two laser beams inspected about 45% of the SPR exposed area (0.42 cm^2^). The sensograms are given as the average Δ*θ* evaluated over the two inspected areas, along with the relative standard deviation, shown as a grey shadow. All experiments were performed in duplicate, on two different SPR slides.

The SPR flow-through cell had a volume of 100 μL and comprised a high refractive index prism coupled to the sensor slide, made of an optical glass covered by a semi-transparent film of thermally evaporated gold (∼50 nm) deposited on a chromium adhesion layer (∼2 nm). Before use, the slides were dip cleaned in an NH_4_OH/H_2_O_2_ aqueous solution (1 : 1 : 5 v/v) at 80–90 °C for 10 min; rinsed with water afterwards, dried with nitrogen, and treated for 10 min in a UV-ozone cleaner. The cleaned SPR slide was then mounted into the SPR sample holder and physisorption was accomplished through static injection of the anti-S1 antibodies (100 μg mL^−1^) in PBS, at pH 7.4 and ionic strengths of 15 mM, 163 mM, and 300 mM. The sensing experiments were performed by exposing the SPR slide coated with physisorbed anti-S1 (100 μg mL^−1^, 163 mM, pH 7.4) to S1 protein standard solutions with concentrations ranging from 1 nM to 0.6 μM. The negative control experiments to evaluate the assay and noise level and selectivity were carried out using a BSA (100 μg mL^−1^) coated SPR slide, exposed to S1 protein standard solutions with concentrations ranging from 1 nM to 0.6 μM. The selectivity of the assay was evaluated by computing the ratio between the angle-shifts measured for the negative control and sensing experiments respectively, Δ*θ*_NC_/Δ*θ*_Sens_. Moreover, the LOD of the assay was evaluated according to the IUPAC definition,^24^ as the projection on the concentration axis of the average level of the negative control experiment plus 3 times its standard deviation, through the calibration curve (Δ*θ vs.* S1 concentration) obtained by regression. To this end, the dose–response curve registered with an anti-S1 coated SPR slide was fitted using a 4-parameter logistic calibration model, mathematically equivalent to the Hill equation (*vide infra*).^25^

Atomic force microscopy (AFM) images were recorded in semi-contact mode (AFM mod. NTEGRA Spectra, NT-MDT, Moscow, Russia) with Si probes (mod. RTESPA-300, Bruker, resonant frequency of 300 kHz, force constant of 40 N m^−1^ and nominal tip radius of 8 nm). The anti-S1 films were physisorbed onto n-doped silicon substrates with a thermally grown 300 nm thick SiO_2_ layer on top. The cleaning procedures consisted of the following sequential steps: an ultrasonic bath in acetone, 2-propanol, and deionized water for 10 min, drying in a nitrogen flux. An adhesion promoter layer of titanium (5 nm) and a gold layer (50 nm) were deposited by electron-beam evaporation (0.1 Å s^−1^, chamber pressure < 10^−6^ mTorr). The gold electrodes were then cleaned of organic residues using a freshly prepared piranha solution, *i.e.*, a mixture of sulfuric acid and hydrogen peroxide (3 : 1 v/v), rinsed with HPLC-grade water, and dried in a nitrogen flux. The anti-S1 patterned bio-functionalization encompassed the following steps: (i) protection of one half of the gold surface using a polymeric mask; (ii) deep coating in a solution of anti-S1 100 μg mL^−1^ in PBS at pH 7.4 and ionic strengths of 15 mM, 163 mM and 300 mM; (iii) mask removal, leaving a dense layer of anti-S1 antibodies on one half of the sample.^26^ AFM images on 5 × 5 μm^2^ areas at the edge across the Au/anti-S1 interface were recorded. The scanning rate was typically 0.3 Hz, and the pixel resolution 0.5 nm or 1 nm per pixel. The grain density was calculated by counting the number of grains per unit area using the Grain Analysis tool of the software Image Analysis, setting a grain diameter threshold of 25 nm.

## 3. Results and discussion

The physisorption of anti-S1 capture antibodies on the sensor slide was carried out by exposing the gold surface to a 100 μg mL^−1^ anti-S1 PBS solution for approximately an hour. PBS at *i*_s_ of 163 mM and pH 7.4 was chosen to mimic physiological conditions.^27^ The deposition process was monitored *in-situ* by measuring the transient shift of the *θ* angle. This mirrors the changes in the dielectric function at the gold/PBS interface, which is quantitatively correlated with the thickness of the biolayer deposition. To this end, the SPR slide was positioned in the sample holder and the physisorption of the antibodies started immediately after the static injection of 100 μL of the anti-S1 PBS solution. After 1 hour the cell was rinsed by injecting 100 μL of PBS. Two sensograms were measured, probing two different regions of the layer, and the average SPR angular variations (Δ*θ*) *versus* time shown in Fig. 1b, evaluated over the two traces, are shown along with the relative standard deviation (gray shadows). The two traces were consistently very similar, with a maximum variation of about 4%, proving the high degree of homogeneity of the anti-S1 capture antibody layer. Upon injection of the PBS anti-S1 solution, a rapid increase in the *θ* angle was registered, proving that a physisorbed film of anti-S1 had already formed on the SPR slide after a few minutes. To quantify the minimum time required to complete the physisorption process, the measured traces were fitted with eqn (1), a kinetic model based on a double exponential function:^28^

1 Δ*θ*(*t*) = Δ*θ*_1_(1 − e^−t/*τ*_1_^) + Δ*θ*_2_(1 − e^−*t*/*τ*_2_^)

where the value Δ*θ*_1_ + Δ*θ*_2_ = 0.69° can be used to estimate the saturation level of the measured trace. The angle variation Δ*θ* as a function of time varies with two apparent association rate constants, *k*_on1_ = 1/*τ*_1_ and *k*_on2_ = 1/*τ*_2_, taking into account the biphasic association occurring at high antibody concentrations.^29^ The two time-constants are *τ*_1_ = 57 s and *τ*_2_ = 12 min.

As is apparent, the first phase, characterized by faster kinetics, is relevant to film deposition and in a time frame of 19 minutes 95% surface coverage is achieved. The second phase has slower kinetics and can be associated with a much slower deposition that most likely takes place when the first protein layer has already been physisorbed. It has already been postulated that a slower binding rate to the sensor surface may arise from steric constraints and electrostatic conditions imposed by the binding of adjacent physisorbed molecules.^29^

The surface coverage of anti-S1 physisorbed on the gold surface was quantitatively assessed, as customary in SPR experiments, by means of de Feijter's equation:^30^2
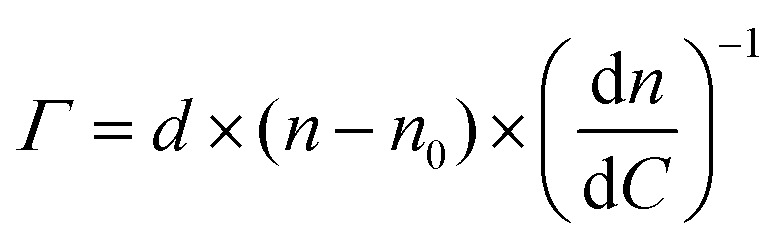
where *Γ*, expressed in ng cm^−2^, is the surface coverage, *d* is the thickness of the biolayer deposited on the gold surface, expressed in nm, (*n − n*_0_) is the difference between the refractive index of the adlayer and that of the bulk medium, while d*n*/d*C* is the specific refractivity of the adsorbed biolayer. Deriving this further to account for the instrument response, gives:

3 (*n* − *n*_0_) = Δ*θ* × *k*

where *k* is the wavelength-dependent sensitivity coefficient, and Δ*θ* is the experimental angular shift of the plasmon peak. For a *λ* = 670 nm laser beam and thin protein layers (*d* < 100 nm), the following approximations hold true: d*n*/d*C* ≈ 0.182 cm^3^ g^−1^ and *k* × *d* ≈ 1.0 × 10^−7^ cm deg.^31^ Therefore, under these assumptions and by substitution of eqn (3) into eqn (2), the surface coverage *Γ* can be estimated using the experimental angular shift, as:

4 *Γ* = Δ*θ* × 550 [ng cm^−2^]

The experimental angular shift (Δ*θ*) inserted into Eqn 4 is the one gathered 30 minutes after the PBS washing step. The average Δ*θ* was indeed as high as 0.68 ± 0.03 deg. According to eqn (4), an anti-S1 surface coverage of 374 ± 16 ng cm^−2^ has been estimated, corresponding to (1.50 ± 0.06) × 10^12^ molecules per cm^−2^, where the error bars have been computed as the propagation error of the surface coverage. This is in line with the very high-density layer deposited by more time-consuming covalent binding approaches.^16,32–34^

**Fig. 2:**
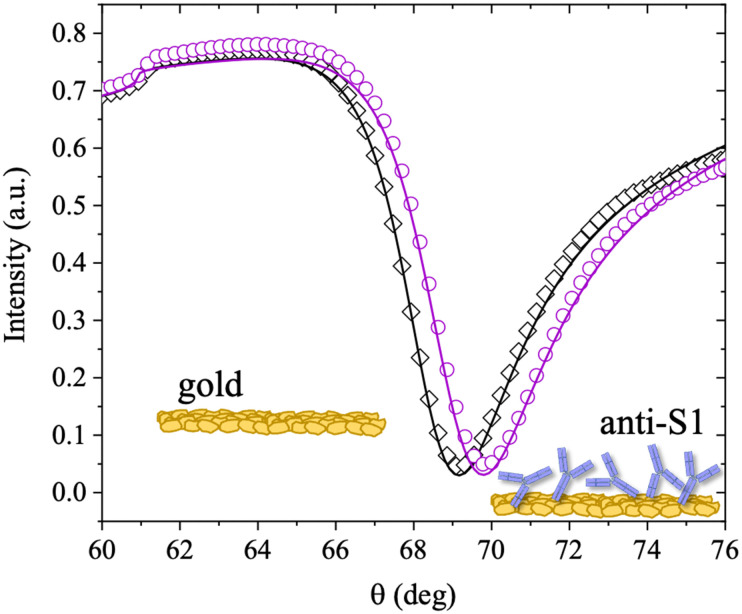
SPR angular reflectivity curves measured at 670 nm for the clean gold surface (black squares) and the physisorbed anti-S1 (purple circles) in PBS; solid lines are the fitted curves (see text for details).

The anti-S1 layer thickness was further investigated by taking advantage of the multi-parametric SPR data collected over the whole angular range (60.00°–76.00°) inspected during the deposition, with an angular resolution of 0.01°. Fig. 2 shows the SPR angular reflectivity curve (reflected intensity) *vs. θ* encompassing the plasmonic peak^14^ before (black squares) and after (purple circles) anti-S1 physisorption. The plasmon peaks shown in Fig. 2 refer to the data at *t* = 0 and *t* = 90 min in Fig. 1b.

The curves were simulated with a multilayer model based on the Fresnel equation using Winspall 3.02 software.^35^ The thicknesses and apparent refractive indexes of the bare slide were estimated first and used as a reference to simulate the thickness change upon anti-S1 physisorption. The thicknesses and optical parameters returned from the simulation of the SPR curves are summarized in Table 1.

**Table 1:** Optical parameters used for the simulations with Winspall 3.02 software of the SPR curves in Fig. 2. The parameters are estimated as the average values and relative standard deviations of 4 traces

Layer	Thickness, *d* (nm)	Refractive Index, *n*	Extinction Coefficient, *k*
Glass BK7	0	1.52	0
Cr	1.8 ± 0.2	3.12 ± 0.05	3.6 ± 0.3
Au	48 ± 2	0.17 ± 0.03	3.79 ± 0.02
Anti-S1	12 ± 1	1.38 ± 0.01	0
PBS	0	1.33	0

The values obtained for the bare Cr/Au coated slides are in very good agreement with the nominal thicknesses declared by the SPR slide provider and with the relevant optical parameters found in the literature.^36^ This proves the reliability of the simulation approach used. The resulting value for the thickness of the anti-S1 layer is 12 ± 1 nm, where the error bar was estimated as the relative standard deviation of the thicknesses simulated on two different replicates and four different sampled areas. An antibody such as anti-S1 holds a y-shaped structure, with typical dimensions of about 14.5 nm × 8.5 nm × 4.0 nm, with the binding sites separated by 13.7 nm.^37,38^ The simulated thickness of the disordered physisorbed layer is compatible with a single monolayer of antibodies. To further validate the model, an independent estimate of the biolayer thickness is provided, by evaluating the number of anti-S1 physisorbed on the surface from the surface coverage previously estimated. The number of anti-S1 on the slide surface area (0.42 cm^2^) is (6.3 ± 3) × 10^11^ molecules, while the theoretical coverage of one layer of all standing antibodies in the slide surface area can be estimated by simple geometrical considerations to be about 7 × 10^11^ molecules. The two figures are comparable and in good agreement with the thickness computed with the Winspall simulations.

**Fig. 3:**
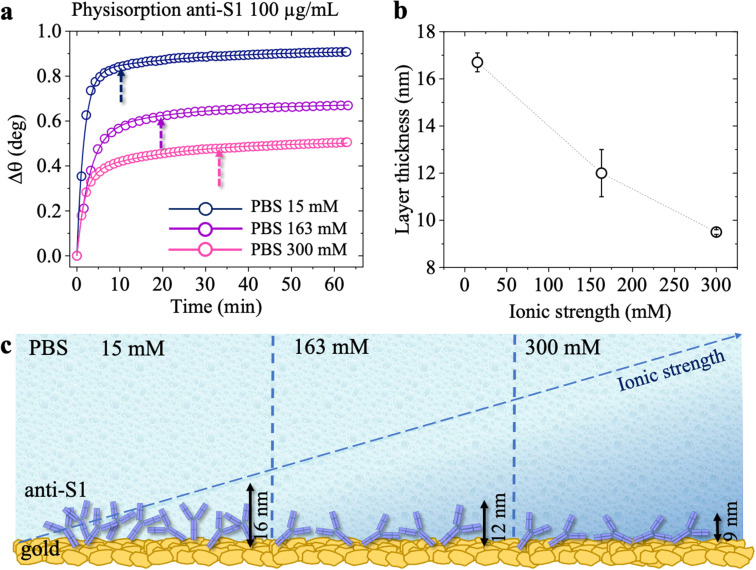
(a) SPR sensograms (Δ*θ vs*. time) of the physisorption of anti-S1 on the SPR gold slide in PBS buffer at different ionic strengths. The hollow circles refer to physisorption in PBS solution at *i*_s_ 15 mM (blue), 163 mM (purple) and 300 mM (magenta). The pH in all cases is kept constant at 7.4. The full lines are the fittings of the traces measured at the different ionic strengths. The dashed arrow indicates the minimum time required for the physisorption to reach 95% surface coverage. (b) Average values of the simulated thickness values. Error bars are the relative standard deviation of simulations performed on two different sampled areas. (c) Schematic illustration of anti-S1 physisorption onto the gold SPR slide at different ionic strengths.

The surface coverage of physisorbed antibodies was also studied as a function of the ionic strength of the anti-S1 PBS solution. The physisorption of the antibodies was monitored by static injection into the SPR cell on three different SPR slides of 100 μL of anti-S1 capture antibody PBS solutions (pH 7.4) at different ionic strengths of 15 mM, 163 mM and 300 mM, while keeping the anti-S1 concentration constant (100 μg mL^−1^). The relevant sensograms are provided in Fig. 3a. The fitting of the sensograms with eqn (1) gives the time to reach 95% surface coverage. The physisorption is accomplished in 8 minutes in a solution with an ionic strength of 15 mM but it becomes much longer (30 minutes) at an ionic strength of 300 mM. The anti-S1 surface coverage was also quantitatively assessed with de Feijter's equation, returning the following values: (1.97 ± 0.04) × 10^12^ molecules per cm^2^ at *i*_s_ = 15 mM, (1.50 ± 0.06) × 10^12^ molecules per cm^2^ at 163 mM and (1.18 ± 0.01) × 10^12^ molecules per cm^2^ at 300 mM. Therefore, the increase in the ionic strength from 15 mM to 300 mM produces a time deposition increase of about 70% and a reduction in the anti-S1 surface coverage of about 40%. The assessment of the anti-S1 layer thicknesses was also investigated by analyzing the reflected intensity *vs. θ* data collected over the whole angular range (60.00°–76.00°), with Winspall 3.02 software. The resulting values for the thicknesses at ionic strengths of 15 mM, 163 mM and 300 mM are (16.7 ± 0.4) nm, (12 ± 1) nm, and (9.5 ± 0.1) nm, respectively. Therefore, by increasing the ionic strength from 15 mM to 300 mM, the average biolayer thickness decreases by 43%. Moreover, the simulated average thicknesses of the physisorbed layer at different ionic strengths are compatible with a single monolayer of antibodies deposited on the gold slides. However, at 15 mM, the computed effective thickness might be consistent mostly with an end-on orientation of the molecules, while the increase in the ionic strength results in an effective thickness compatible with a mainly lying-flat configuration of the antibodies.^39,40^

**Fig. 4:**
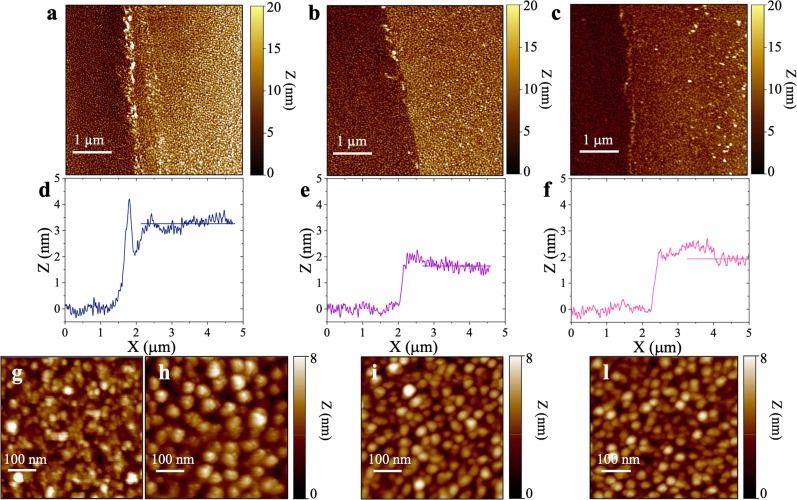
Topographic semi-contact AFM images recorded in air at the interfaces between the bare Au (leftmost) and the physisorbed anti-S1 covered (rightmost) areas of a gold surface exposed to PBS antibody solutions with a constant pH = 7.4 and ionic strengths of (a) 15 mM, (b) 163 mM, and (c) 300 mM. Corresponding averaged profiles across the interfaces for samples relevant to physisorption at different ionic strengths of (d) 15 mM, (e) 163 mM, and (f) 300 mM. Topographic semi-contact 500 nm × 500 nm (1000 × 1000 pixels) AFM images of (g) pristine gold surface and anti-S1 physisorbed on gold electrode in PBS solutions of (h) 15 mM, (i) 163 mM, (l) 300 mM.

Fig. 4 shows the topographical AFM images recorded in air at the interfaces between the bare (leftmost) and the physisorbed anti-S1 covered (rightmost) areas of a gold surface exposed to PBS antibody solutions with a constant pH = 7.4 and ionic strengths of 15 mM (Fig. 4a), 163 mM (Fig. 4b), and 300 mM (Fig. 4c). The corresponding averaged profiles across the interfaces, given in Fig. 4d, e, f, show that the film edge thickness also decreases with ionic strength in the dried samples used for AFM measurements. At low ionic strength (15 mM) the average thickness is about (3.3 ± 0.2) nm (Fig. 4d), while at higher ionic strengths (163 mM, 300 mM), the edge thickness decreases to (1.6 ± 0.1) nm (Fig. 4e) and (1.8 ± 0.1) nm (Fig. 4e), respectively. Importantly, the edge thicknesses measured with AFM characterization in air are lower than those returned by the SPR analysis performed directly in the liquid. Such evidence compares well with a recent study reporting an increase in the thickness of Alzheimer paired helical filament layers as measured by AFM in liquid with respect to a dry environment.^41^Fig. 4g shows that the gold surface is characterized by the presence of 10–20 nm grain-type features and roughness of 0.89 nm, typical for e-beam evaporated gold thin films. On the other hand, the AFM image of Fig. 4h shows that at 15 mM the biolayer surface is characterized by 26 ± 11 nm grain-type features, that can be ascribed to protein clusters. Their surface density is ∼604 grains per μm^2^ and the roughness is 1.18 nm. Therefore, based on the anti-S1 surface coverage determined by SPR analysis, each grain-type feature comprises about 32 antibodies. At higher ionic strengths of 163 mM (Fig. 4i) and 300 mM (Fig. 4l), the protein cluster size reduces to 17 ± 9 nm grain-type features, and their surface density increases to ∼1408 grains per μm^2^ and ∼2140 grains per μm^2^, respectively. This results in a number of anti-S1 antibodies per grain-type feature of about 11 and 6, respectively. Moreover, at higher ionic strength the average roughness is also reduced to 0.98 nm, suggesting a preferred lying-down configuration of the antibodies.^42^ Indeed, it has been recently proved that at higher ionic strength the protein–protein electrostatic interactions are decreased, due to screened electrostatic interactions.^43^ This results in an adsorption pattern characterized by smaller and more homogeneous protein aggregates with a significant reduction in surface roughness, in agreement with the SPR and AFM analyses.

The AFM analysis supported by SPR characterization is consistent with a model predicting that each molecule occupies a larger surface area when deposited from a solution of higher ionic strength.^39^ Indeed, electrostatic interactions are the driving forces in protein adsorption.^39,44,45^ This holds even more true at low ionic strength, where the electrostatic interactions are the dominant factor in controlling the physisorption of antibodies, enhancing the formation of protein clusters, thus increasing the surface roughness along with the biolayer thickness.^43^ In contrast, Monte Carlo simulations of protein adsorption have recently shown that the lying-flat orientation of the antibodies is favored at high ionic strength, being screened by electrostatic interactions,^39^ in agreement with SPR.

The binding efficacy of the physisorbed anti-S1 capture antibodies was validated through an SPR direct label-free assay of the spike S1 target antigen, also after aging of the capture layer. The anti-S1 coated SPR sensor slide was tested against the binding of the S1 protein spanning the overall range of concentrations of 1 nM to 600 nM, typical for an SPR label-free assay experiment.^46^

**Fig. 5:**
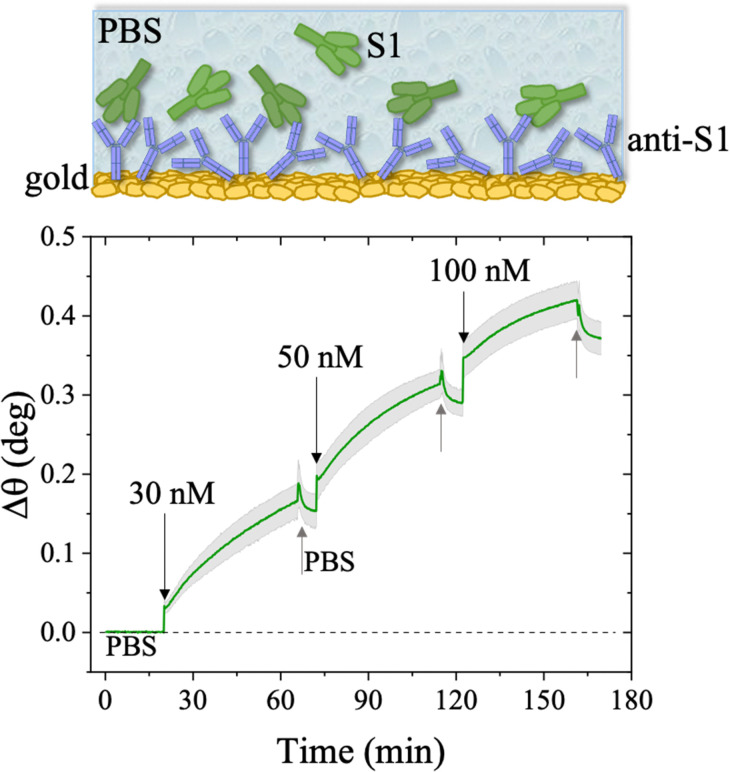
Sensogram of the SPR gold slide with the anti-S1 physisorbed film exposed to different concentrations of S1 affinity antigen, stored in PBS. The experiment was performed in the S1 range of concentration 30 nM–100 nM. The sensogram (green line) refers to the average SPR trace registered while sampling the anti-S1 layer in two different points, 3 millimeters apart. The grey shadow is the relative standard deviation. The black arrows indicate the times of the injections into the SPR flow-through cell for S1 standard solutions while the grey arrows indicate the PBS washing steps.

The first S1 sensogram measured with a physisorbed anti-S1 SPR slide stored in PBS, is shown in Fig. 5, where the range 30 nM–100 nM was explored. The assay was carried out by injecting S1 solutions into PBS at different concentrations. Each solution was left to interact with the anti-S1 functionalized surface for 40 minutes (Fig. 5), the typical timeframe to reach a stable SPR signal in the manual injection mode. Indeed, based on previous studies, a stable signal during incubation steps, corresponding to 95% of the plateau, is typically recorded after 20 minutes.^47^ Upon stabilization, the unbounded S1 antigens were removed by rinsing with PBS buffer solution. The signal after each rinsing was compared to the baseline, acquired in PBS buffer solution, taken as the zero-level signal in the sensogram. Also in this case, the exposed sensing area was sampled in two different points, and the average Δ*θ* (green signal) along with the relative standard deviation (grey shadow) are reported in Fig. 5; the two traces show very similar features, as proven by the maximum standard deviation of 0.02° comparable to the instrumental error. This proves the uniformity of the capture properties of the physisorbed anti-S1 layer. Relevantly, the binding of S1 antigens produces an SPR signal as high as 0.37° ± 0.02° in the investigated range of concentration. The signal is comparable with a similar SPR immunoassay encompassing covalent binding of the capture antibodies through a mixed self-assembled monolayer (SAM).^16^

**Fig. 6:**
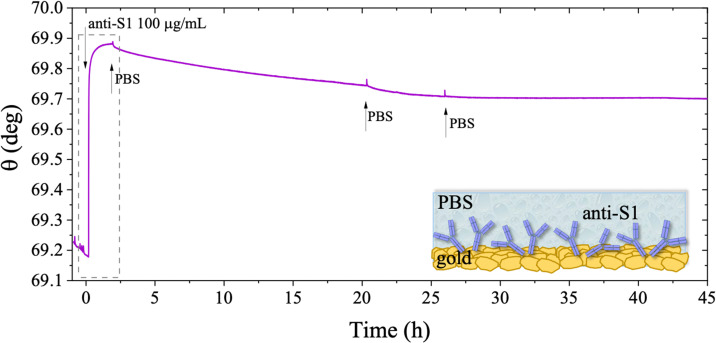
SPR trace measured on a gold slide covered with anti-S1 film physisorbed from PBS and kept in buffer solution afterwards. The sensogram is the average signal gained from two simultaneously inspected areas on the same sensor slide. The SPR optical signal is recorded for 45 hours to accomplish the real-time monitoring of the anti-S1 stability in PBS (*i*_s_ 163 mM, pH 7.4). Black arrows indicate the times of the injections into the SPR cell of the antibody solution while the gray arrows indicate the bare PBS injections. The insert shows the first 1.5 hours of analysis for the physisorption of anti-S1 film on gold and the subsequent washing step.

**Fig. 7:**
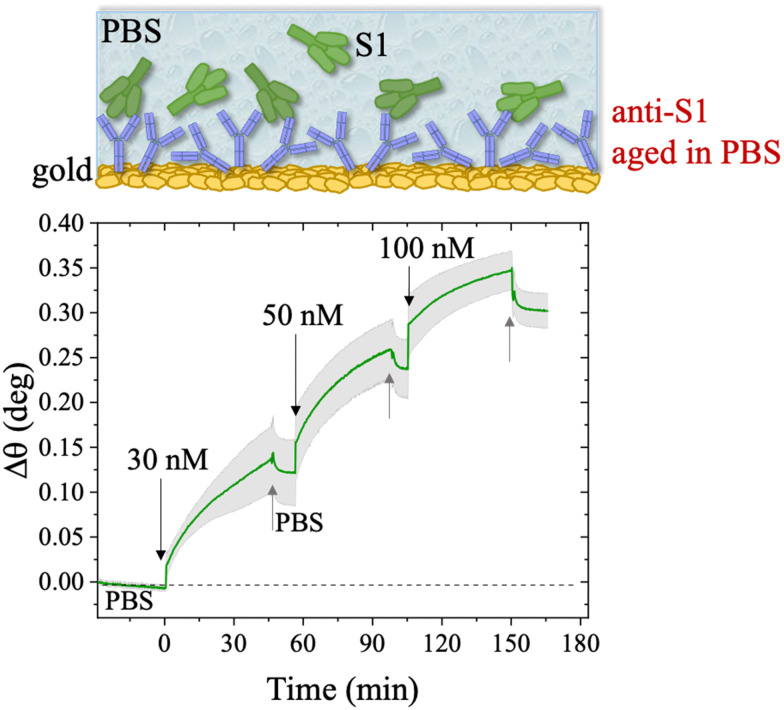
Sensogram of the SPR gold slide with anti-S1 physisorbed after prolonged storage in PBS for two weeks at 4 °C. The S1 concentration used in the experiment was in the range 30 nM–100 nM. The sensogram (green line) is reported as the average trace among those collected in two different sampled points of the anti-S1 layer, 3 millimeters apart. Black and gray arrows specify the times of the injections into the SPR flow-through cell for S1 standard solutions and PBS washing steps, respectively.

The long-term stability of the physisorbed proteins was also investigated in depth, under different storage conditions. First, the stability of the physisorbed biolayer, after storage in PBS for 45 hours, was assessed, proving that physisorption of the protein on gold can lead to a stable immobilization protocol. The average trace recorded for the two sampling areas covered with anti-S1 and exposed to PBS for a prolonged time, is given in Fig. 6. After 45 hours of exposure to the anti-S1 film in bare PBS, a *Γ* of 280 ± 1 ng cm^−2^ surface coverage was measured, corresponding to a highly packed layer of capture antibody comprising (1.13 ± 0.04) × 10^12^ molecules per cm^2^. This returns a figure of the order of 10^4^ proteins per μm^2^, which is considered the limit for the packing of proteins on a surface.^48,49^ In this very prolonged exposure to PBS, only a 25% overall desorption was observed. Notably, after 30 hours of storage negligible desorption as low as 0.01% was observed, meaning that an even longer storage time will not produce any significant decrease in terms of surface coverage. Besides, the observed slight desorption of the bio-recognition elements does not produce a reduction in the assay performances (*vide infra*), since trillions of capture antibodies are still available on the sensor surface. The long-term stability of the physisorbed layer was also assessed as the binding capacity of the antibodies against the target antigen, after further storage of the SPR slide in PBS for two weeks. To this end, the biofunctionalized SPR slide was stored in PBS solution for two weeks at 4 °C. The assay for S1 was then performed over the same range of concentrations and the relevant sensogram is given in Fig. 7. Noticeably, the traces recorded upon protein binding after prolonged storage are comparable within one standard deviation with those recorded with the SPR slide tested immediately after physisorption. This proves the excellent long-term stability of the physisorbed deposit, as well as the non-compromised binding capacity of the antibodies. A quantitative comparison between these two assays is summarized in Table 2.

**Table 2:** SPR angular shift (Δθ) of the S1 assay involving a freshly physisorbed anti-S1 layer that is compared to an assay carried out on an anti-S1 layer stored for two weeks in PBS and after 24 hours storage in water (HPLC grade)

S1 concentration (nM)	Δ*θ*°		
	PBS	2 weeks storage in PBS	24 hours storage in HPLC-grade water
30	0.15 ± 0.02	0.12 ± 0.04	0.12 ± 0.02
50	0.29 ± 0.02	0.24 ± 0.03	0.25 ± 0.02
100	0.37 ± 0.02	0.30 ± 0.02	0.34 ± 0.01

**Fig. 8:**
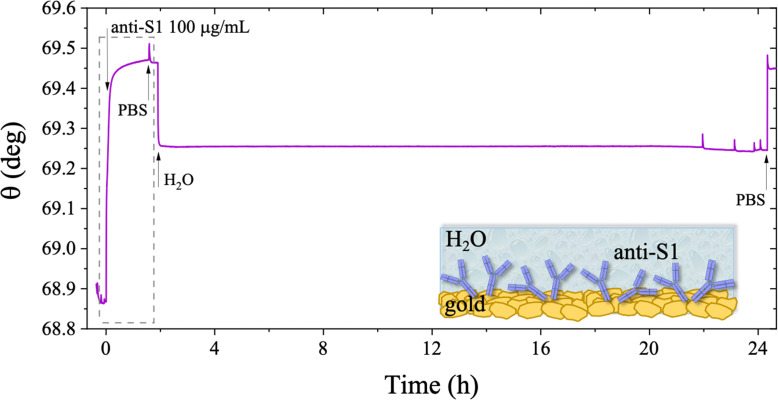
SPR trace measured on a gold slide covered by anti-S1 film deposited from PBS and kept in deionized water (HPLC grade) afterwards. The real-time monitoring of the deposit stability in water is shown for24 hours. The black trace is the average value of the signal measured for two points simultaneously tested on the same sample. Black arrows specify the times of the injections into the SPR cell. The insert shows the first 1.5 hours of the physisorption of anti-S1 film on gold and the subsequent washing step.

**Fig. 9:**
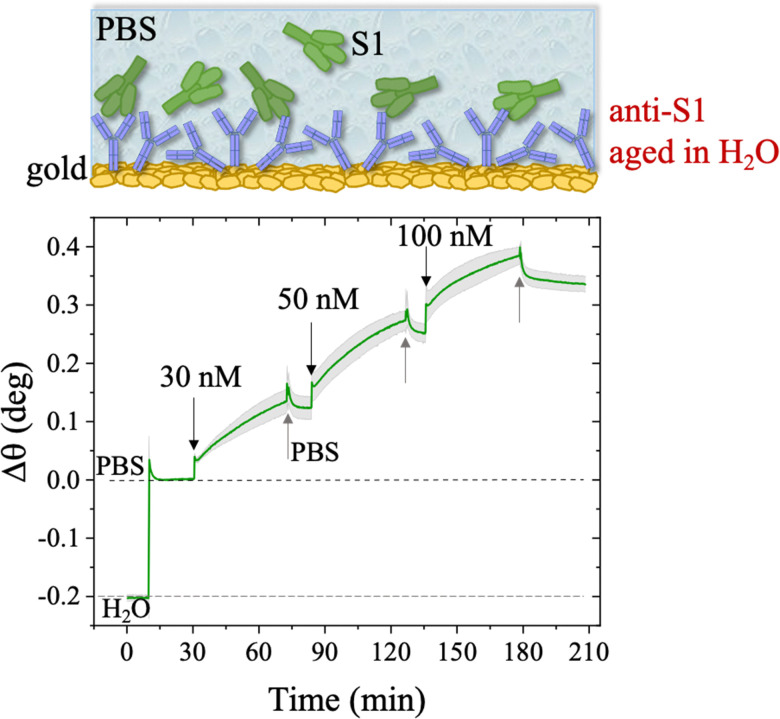
S1 Sensogram of an anti-S1 layer, kept for 24 h in water. The experiment was performed in the S1 range of concentrations 30 nM–100 nM. The green trace is the average of the SPR signals registered by sampling the anti-S1 layer in two different points, 3 millimeters apart, along with the relative standard deviation. Black and grey arrows specify the times of the injections into the SPR flow-through cell for S1 standard solutions and PBS washing step, respectively.

The stability of a physisorbed antibody layer stored in deionized water (HPLC grade) for 24 hours was also investigated, and the corresponding SPR trace is shown in Fig. 8. After deposition of the anti-S1 film in a PBS solution for 1.5 hours, the surface was rinsed with PBS until a stable baseline was recorded. The SPR cell was subsequently filled with deionized water, and kept in contact with the physisorbed anti-S1 layer for 24 hours. The injection of water causes an abrupt decrease in the SPR signal, ascribable to a change in the refractive index of the bulk solution. Afterward, a negligible decrease in the SPR signal was registered during exposure to water, showing extremely good stability. To quantify the stability, the refractive index of the solution in the cell was brought back to the original value, by substituting deionized water with PBS. The surface coverage measured before water injection was 326 ± 16 ng cm^−2^, corresponding to (1.31 ± 0.06) × 10^12^ molecules per cm^2^. After overnight exposure in water, the coverage was still as high as 318 ± 22 ng cm^−2^ corresponding to (1.2 ± 0.1) × 10^12^ molecules per cm^2^. Remarkably, a highly negligible anti-S1 desorption below 3% was registered after 24 h in deionized water, showing that the anti-S1 antibodies are more prone to remain segregated on the gold surface, instead of redissolving in deionized water.^50,51^ The capture efficacy of the biorecognition elements after storage in deionized water was investigated to rule out major loss of bioactivity due to structural rearrangements occurring in the water environment.^52–54^ Indeed, the degradation mechanism of proteins has been extensively studied and represents a main drawback when developing a performing assay.^55,56^ The sensogram of an anti-S1 layer stored in water is given in Fig. 9 and it shows features that are comparable within one standard deviation to those registered for the SPR slide stored in PBS.

**Fig. 10:**
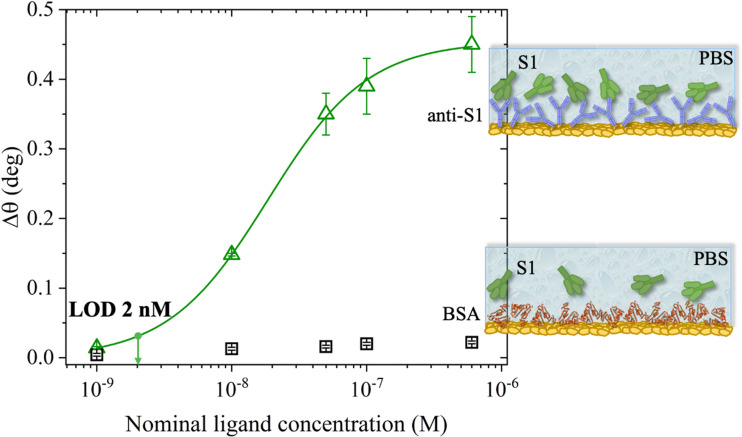
S1 protein SPR assay carried out in PBS standard solutions with concentrations in the 1 nM–0.6 μM range. The SPR angle-shift is taken as the assay response. The green hollow triangles are the data measured with the SPR slide coated with physisorbed anti-S1 antibodies, while the black hollow squares are those measured in the negative control experiment that encompasses a bare BSA coated SPR slide. Error bars over two replicates are set as one standard deviation. The modeling (green solid curve) was performed with an analytical model based on a 4-parameter logistic equation. The LOD, taken as the average of the control experiment data (noise level) plus three times the standard deviation, is equal to 2 nM.

The SPR angular shifts recorded upon exposure to three subsequent S1 standard solutions (30 nM, 50 nM and 100 nM) are reported in Table 2 for both the biofunctionalized SPR slides stored in PBS (*i*_s_ = 163 mM, pH = 7.4) and in water (*i*_s_ = 5 μM, pH = 5.5, HPLC-grade) environment. The data summarized in Table 2 clearly show that physisorbed anti-S1 antibodies retain their full biological functionality, even when the layer is stored for several hours in a deionized water environment. This aspect should not be taken for granted, as proteins in nature are never found in pure water and they must be stored in an appropriate temperature and pH range to retain activity.^57^ Nonetheless, there are a number of high-performance biosensing applications where the sensing experiment is performed in pure water, resulting in a Debye length of up to 100 nm, to maximize the sensitivity of the assay.^58–61^ In fact, the Debye length, being the scale over which mobile ions screen out electric fields of fixed charges on the biomolecules, is a fundamental parameter for enhancing the sensitivity and limit-of-detection in biosensors and it is inversely proportional to the square root of ionic strength.^62^ Hence, a typical approach to increase the Debye length is to reduce the electrolyte ion concentration. This SPR study not only allowed us to prove the long-term stability of physisorbed antibodies stored in pure water, but also that their capture efficacy against the target antigen is fully retained even under storage in non-physiological conditions. The selectivity of the assay has been addressed through a negative control experiment registered by exposing a bovine serum albumin (BSA) coated SPR slide to S1 proteins. The average Δ*θ vs.* the concentration of nominal S1 proteins, ranging from 1 nM to 0.6 μM, registered with the anti-S1 and BSA-coated SPR slides are shown in Fig. 10. Each Δ*θ* reported in Fig. 10 at increasing concentrations of S1 standard solutions, was evaluated as the SPR angle difference between the signal after the PBS washing step of the incubated slide and the baseline. The green hollow triangles and black hollow squares refer to the average of the exposure to S1 analyte standard solutions evaluated with two replicates of the anti-S1 and BSA-coated SPR slides, respectively. The full green line represents the dose-curve fit of the sensing experiment, based on the following 4-parameter logistic (4PL) calibration model:^63,64^5
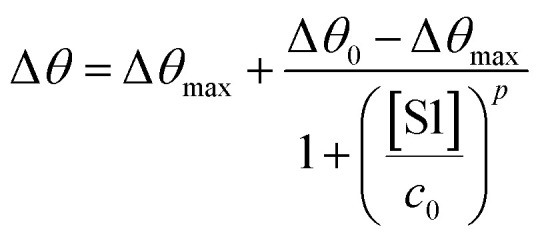
where [S1] is the concentration of nominal S1 proteins, while Δ*θ* is the SPR angle shift. Moreover, Δ*θ*_0_ and Δ*θ*_max_ are the minimum and maximum SPR angle shifts registered in the dose–response curve, defining the assay dynamic range,^65^ while *c*_0_ is the inflection point where the curvature changes sign, marking the analyte concentration where a response decrease of 50% occurs.^25^ Moreover, parameter *p* controls the symmetry of the distribution. The fitting procedure was repeated several times, and the coefficients were adjusted depending on the residual errors in the previous iteration.

The selectivity of the biosensing platform has been successfully demonstrated, as the negative control experiment involving the BSA coated SPR slide showed a maximum angle-shift below 0.02° ± 0.01°, being only 2% of the signal registered with the sensing experiment and falling within the instrumental error range. Hence, the selectivity of the assay has been quantitatively addressed as the ratio between the angle-shift measured for the negative control and sensing experiments, respectively, resulting in a value as low as Δ*θ*_NC_/Δ*θ*_Sens_ = 0.04 ± 0.01. Moreover, the limit of detection (LOD) level has been evaluated as the average SPR angle-shift of the negative control experiment plus three times its standard deviation. Remarkably, an LOD as low as 2 nM has been achieved. Therefore, the SPR assay developed herein, encompassing a bare physisorbed layer of anti-S1 capture antibodies, is among the best-performing SPR assays reported so far in terms of LOD and selectivity,^19,20,66^ being also cost-effective, more rapid than other immobilization techniques, and avoiding the use of coupling reagents. Indeed, an assay encompassing an SPR slide modified with a short hetero-functional poly(ethylene glycol) (PEG) linker, HS-PEG6-COOH, followed by covalent coupling with VHH-72-Fc nanobodies using a standard amide coupling procedure (NHS/EDC) to detect SARS-CoV-2 S1 proteins, recently achieved an LOD of 10 nM and a selectivity ratio Δ*θ*_NC_/Δ*θ*_Sens_ of about 0.13, one order of magnitude higher than that achieved in the present study.^19^

## 4. Conclusions

SPR is herein proposed to assess the long-term stability and high-performance sensing of a physisorbed antibody layer. The study involved a physisorbed SARS-CoV-2-Spike-S1 antibody (anti-S1) tested against S1 in PBS solutions (pH 7.4 and ionic strength of 163 mM) mimicking physiological conditions. The physisorption was carried out in an SPR cell on a gold-coated sensor slide and monitored *in situ*. A modelling of the sensogram (plasmon angular shift, Δ*θ vs.* time) showed that in 19 minutes, 95% surface coverage was accomplished, resulting in (6.3 ± 3) × 10^11^ molecules on the 0.42 cm^2^ sensing surface. Computation of the deposit thickness was also undertaken, relying on SPR reflectivity curves *vs. θ*, collected over the whole angular range inspected during the deposition. The resulting value for the effective average thickness of the anti-S1 layer is 12 ± 1 nm. Considering that the physisorption was proven to be homogeneous by a systematic two-point inspection of the surface, the derived thickness is compatible with a single monolayer of nearly end-on antibodies deposited on the gold surface. The antibodies are also likely to be mostly arranged upright, as discussed in studies on protein–protein electrostatic interactions.^39,40,45^ The estimated anti-S1 surface density of (1.50 ± 0.06) × 10^12^ molecules per cm^2^ is close to the highest possible packing of a protein on a surface, 10^4^ μm^2^,^48^ and is comparable to what has been achieved with more sophisticated and time-consuming biofunctionalization procedures involving the chemical conjugation of the recognition elements to the gold surface.^19,32,67^ The effect of the PBS solution ionic strength on physisorption of the antibodies was also thoroughly investigated. To this end, the anti-S1 capture antibodies were deposited from PBS solutions (pH 7.4) at ionic strengths of 15 mM, 163 mM, and 300 mM. The data show that lowering the ionic strength from 300 nM down to 15 mM produces an enhancement in the anti-S1 surface coverage of about 40%, along with an increase in the protein adlayer thickness, reaching a maximum of (16.7 ± 0.4) nm, compatible with end-on antibody orientation. Moreover, a deposition time as fast as 8 minutes is required to reach 95% surface coverage obtained at an ionic strength of 15 mM, while the deposition time increases to 30 minutes when the anti-S1 physisorption occurs at an ionic strength of 300 mM. The AFM morphological characterization of the anti-S1 antibodies physisorbed at different ionic strengths shows that at higher ionic strength the lateral protein–protein electrostatic interactions are decreased. This results in an adsorption pattern characterized by smaller and more homogeneous protein aggregates while increasing the solution ionic strength gives a significant reduction in surface roughness, in agreement with the SPR analysis. All this clearly supports the pivotal role played by electrostatic interactions in controlling the physisorption of antibodies. The long-term stability of the physisorbed proteins was also investigated, under different storage conditions. Notably, SPR sensing proves the long-term stability of physisorbed antibodies stored not only in PBS (pH = 7.4 and ionic strength 163 mM) but also in non-physiological deionized water, along with proving that the S1 capture efficacy is fully retained. Moreover, with the SPR direct assay an LOD as low as 2 nM and a selectivity ratio of 0.04 were achieved, competitive with other optical sensing platforms, which rely on chemical grafting of capture antibodies.^19,20^ This study can pave the way towards ultrasensitive biosensing applications, encompassing a sustainable, and cost-effective biofunctionalization protocol, also compatible with printing techniques, based on physical immobilization of a biorecognition element on a biosensor transducing interface.

## Conflicts of interest

The authors declare no conflict of interest.

## Supplementary Material
